# Hyperspectral Imaging (HSI) as a new diagnostic tool in free flap monitoring for soft tissue reconstruction: a proof of concept study

**DOI:** 10.1186/s12893-021-01232-0

**Published:** 2021-04-30

**Authors:** Lukas H. Kohler, Hannes Köhler, Simon Kohler, Stefan Langer, Rima Nuwayhid, Ines Gockel, Nick Spindler, Georg Osterhoff

**Affiliations:** 1 Department of Orthopedic, Trauma and Plastic Surgery, Leipzig University Hospital, Liebigstraße 20, 04103 Leipzig, Saxony Germany; 2 Innovation Center Computer Assisted Surgery (ICCAS), University of Leipzig, Leipzig, Saxony Germany; 3 Department of Visceral, Transplant, Thoracic and Vascular Surgery, Leipzig University Hospital, Leipzig, Saxony Germany

**Keywords:** Hyperspectral imaging, Reconstructive surgery, Monitoring, Imaging, Flap surgery

## Abstract

**Objectives:**

Free flap surgery is an essential procedure in soft tissue reconstruction. Complications due to vascular compromise often require revision surgery or flap removal. We present hyperspectral imaging (HSI) as a new tool in flap monitoring to improve sensitivity compared to established monitoring tools.

**Methods:**

We performed a prospective observational cohort study including 22 patients. Flap perfusion was assessed by standard clinical parameters, Doppler ultrasound, and HSI on t0 (0 h), t1 (16–28 h postoperatively), and t2 (39–77 h postoperatively). HSI records light spectra from 500 to 1000 nm and provides information on tissue morphology, composition, and physiology. These parameters contain tissue oxygenation (StO2), near-infrared perfusion- (NIR PI), tissue hemoglobin- (THI), and tissue water index (TWI).

**Results:**

Total flap loss was seen in n = 4 and partial loss in n = 2 cases. Every patient with StO2 or NIR PI below 40 at t1 had to be revised. No single patient with StO2 or NIR PI above 40 at t1 had to be revised. Significant differences between feasable (StO2 = 49; NIR PI = 45; THI = 16; TWI = 56) and flaps with revision surgery [StO2 = 28 (p < 0.001); NIR PI = 26 (p = 0.002); THI = 56 (p = 0.002); TWI = 47 (p = 0.045)] were present in all HSI parameters at t1 and even more significant at t2 (p < 0.0001).

**Conclusion:**

HSI provides valuable data in free flap monitoring. The technique seems to be superior to the gold standard of flap monitoring. StO2 and NIR PI deliver the most valuable data and 40 could be used as a future threshold in surgical decision making. *Clinical Trial Register* This study is registered at the German Clinical Trials Register (DRKS) under the registration number DRKS00020926.

## Introduction

The performance of free tissue transfer in the context of soft tissue reconstruction has been performed since the late 1950s using a vascularized intestinal segment for cervical esophagus reconstruction [1]. Due to the continuous optimization of pre-, peri- and postoperative settings, survival rates of free flaps range between 91 and 95% nowadays [2–5]. Despite overall good success rates, vascular complications play a decisive role in postoperative management. Revision surgery due to vascular complications occurs in 5–25% [6–13]. Vascular processes such as microthrombolization, endothelial cell retraction, and vascular spasms lead to a “no reflow” phenomenon, which is irreversible (0% survival rate, n = 15) in terms of free flap salvage when perfusion has been inhibited for more than 12 h in a rabbit model after revascularization. Flap salvage in the correlated groups with revascularization after 1 h (n = 15), 4 h (n = 15) and 8 h (n = 15) was 80–100%. Thus the survival rate is inversely related to the occurrence of ischemia [14, 15].

Vascular monitoring of the transplanted flaps by clinical and instrumental tools is therefore of crucial importance and leads to a better outcome [16]. Monitoring should be rapid, accurate, reliable, and practicable to all flap types. The gold standard of flap monitoring to date is clinical assessment (flap color, capillary refill, tissue turgor, temperature) and handheld Doppler sonography [17]. Several other techniques measure the circulatory flow (implantable Doppler, color duplex ultrasonography, fluorescence angiography, laser Doppler flowmetry) or tissue metabolism and ischemia (Near-Infrared Spectroscopy, microdialysis) [18–23]. So far, however, none of these methods have been able to establish itself in clinical routine since there is a lack of data that demonstrates a reliable, clinical superiority and cost efficiency [17, 18, 24].

A particular challenge is the monitoring of transplanted flaps without a skin island. The skin is missing as a reliable predictor of possible vascular complications. In the case of venous thrombosis, for example, Doppler ultrasound continues giving regular sound feedback and abnormal tissue configuration can be prolonged [25, 26].

Hyperspectral imaging is a new in vivo imaging technique that combines the principles of spectroscopy and imaging in a non-contact fashion to provide information about tissue morphology, composition, and physiology [27]. HSI creates high-resolution images that contribute information about oxygenation or ischemia in superficial tissue layers. Several experimental and clinical studies have shown that HSI delivers reliable data in wounds (diabetic, peripheral arterial occlusive disease, burn) [28–33], oncologic surgery (esophagectomy) [34], intestinal resections [35], left liver resection [36] and maxillofacial surgery [37].

This study aims to show the superiority of the HSI technique over already established monitoring procedures and that it can thus reduce the occurrence of vascular complications.

## Patients and methods

### Study collective

We performed a prospective observational cohort study. Patients aged 18 and older who underwent soft tissue reconstruction using a free flap between March 2019 and January 2020 and had given informed consent were eligible. In total, 22 patients (17 males, five females) with a median age of 55 (26–92) were included. Next to age, we analyzed gender, flap indication, comorbidities, and the hospitalization after surgery (Table 1).

**Table 1:** Baseline data

	No revision	Partial revision	Complete revision
N (22)	16	2	4
Age [year]	52.5 (SD 14)	65.5 (SD 37)	57.5 (SD 12)
Gender [f:m]	3:13	0.2	2.2
Indication			
Trauma	9	2	1
Infection	4	0	2
Cancer	3	0	0
POVD	0	0	1
Hospitalization after surgery (d)	12 (SD 6.6)	11.5 (SD 2.1)	30 (SD 14.5; p = 0.0013)
Comorbidities [n (%)]			
Diabetes	2 (12.5%)	0	3 (75%)
POVD	2 (12.5%)	1 (50%)	1 (25%)
CHD	2 (12.5%)	0	1 (25%)
Arterial hypertonia	6 (37.5%)	0	3 (75%)
Atrial fibrillation	0	0	1 (25%)
Smoking	3 (18.8%)	0	1 (25%)
Cancer history	3 (18.8%)	0	0
Oral anticoagulation	1 (6.3%)	0	1 (25%)

*POVD peripheral occlusive vessel disease, *CHD* chronic heart disease

Flaps included ALT flaps (n = 11), Latissimus Dorsi flaps (n = 4), Deep Inferior Epigastric Artery Perforator (DIEAP) flaps (n = 3), Muscle Sparing Free Transverse Rectus Abdominis Myocutaneus (MS2-TRAM) flaps (n = 2), Parascapular flaps (n = 1) and Rectus Abdominis flaps (n = 1). Both flaps with (n = 18) and without (n = 4) skin islands were included.

The primary endpoint was revision surgery during hospitalization and the primary outcome was the revision rate.

### Flap assessment by clinical and handheld Doppler sonography

Flap perfusion was assessed by standard clinical parameters (flap color, capillary refill, tissue turgor, temperature) regularly due to our clinical standard assessment (every 2 h within the first 24 h and every 4–72 h postoperatively) by handheld Doppler ultrasound (Dopplex D900 Audio Only Doppler, Huntleigh Healthcare Limited, Cardiff, United Kingdom).

### Flap assessment by hyperspectral imaging

**Fig. 1:**
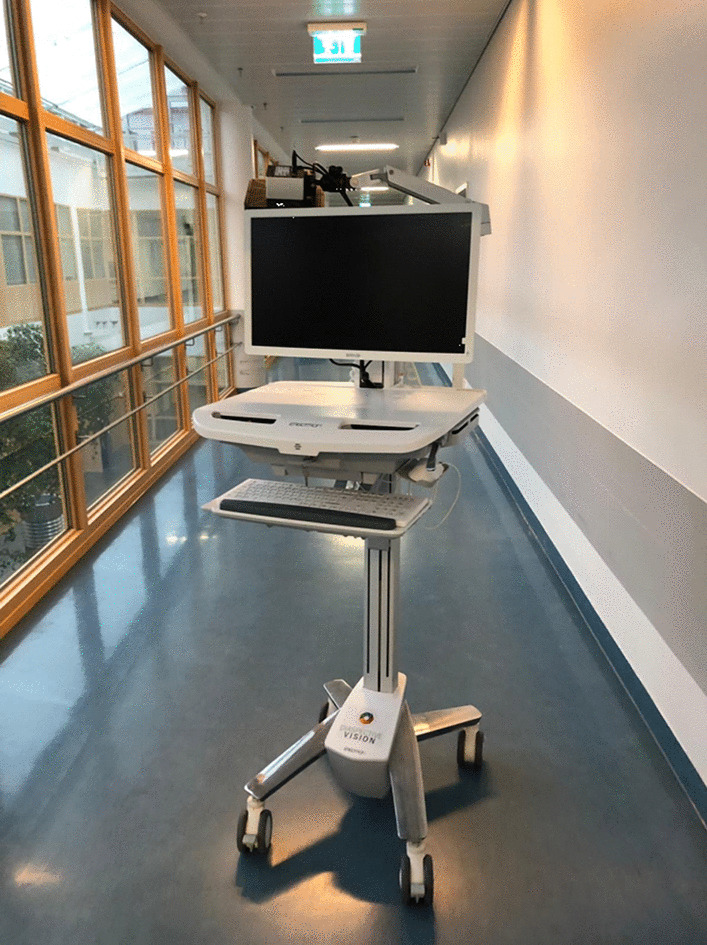
The Hyperspectral Imaging Camera System consisting of a mobile table stand, a swiveling hyperspectral camera including an illumination unit and objective lenses as well as the integrated evaluation software. Picture Copyright© Leipzig University Hospital, Department of Orthopedic, Trauma and Plastic Surgery; Leipzig Saxony, Germany

Next to clinical and Doppler controls, we performed hyperspectral imaging at three fixed points in time (t0 = day of surgery, t1 = first day postoperatively, t2 = second day postoperatively) using the hyperspectral imaging camera and its evaluation software (Fig. 1). For HSI data acquisition the commercially available TIVITA^®^ Tissue camera (Diaspective Vision GmbH, Germany) was used. The system uses a built-in halogen light source to record reflectance spectra from 500 to 1000 nm in 6.4 s. This data is processed in real-time and false-color images represent physiologic tissue parameters in the range from 0 to 100. These parameters were previously described and evaluated by Holmer et al. [38]. Tissue oxygenation (StO2) and near-infrared perfusion index (NIR PI) are more suitable for the assessment of flap perfusion than tissue hemoglobin- (THI) and tissue water index (TWI). Thus, the focus in this work is on StO2 and NIR PI. StO2 provides information about the microcirculation in the most superficial tissue layers (penetration depth 1 mm) whereas near-infrared light has a higher penetration depth (4–6 mm) into the body due to lower absorption by hemoglobin. The field of view (FOV) for all parameters was 21 × 30 cm^2^, which corresponds to the size of a DIN A4 page, and the spatial resolution was 0.56 mm at 630 nm (evaluated with the 1951 USAF resolution test chart at 50 cm object distance). For the measurement process, the hyperspectral camera, which consists of a mobile table stand, a swiveling hyperspectral camera including illumination unit and objective lenses as well as integrated evaluation software, is placed at a distance of 50 cm above the patient in a contactless fashion. The data recorded by the camera is visually processed by the built-in software supplied and made available as false-color images. The entire evaluation takes about 15 s, is contactless and safe. Further analysis was performed by placing three circular regions of interest (ROI) at the center of the proximal third, intermediate, and distal flap part with diameters about 3 cm. Each ROI was assessed separately regarding the clinical parameters, mean StO2, and mean NIR PI.

### Statistical analysis

Continuous data are presented in mean and standard deviation (SD), categorical data in frequencies and percentages. The f-test was performed to check for equal variances and an unpaired two-tailed Student’s t-test was used to detect differences in means of normally distributed data. P-values less than 0.05 were considered significant. All calculations were performed with TIVITA^®^ Suite and Microsoft Excel 2013. For flap survival, a complete or partial revision was counted as an event. The observation period ended after the patient discharge from the hospital.

### Institutional review board number

The study protocol of this study was approved by the institutional ethics committee (reference number 051/19-ek).

## Results

### Baseline data

Complete revision of the flap was necessary in four cases (18.2%) including two Latissimus Dorsi flaps, one Rectus abdominis flap, and ALT flap. Partial revision in two cases (9.1%), both were ALT flaps. The indication for revision was determined by our routine check-ups (clinical, handheld Doppler). Revision surgery in cases with complete flap loss was performed between postoperative day two and eight. One case with partial flap loss was covered with split skin 20 days after initial surgery, the other case was managed in a secondary wound healing setting without the need for revision surgery. None of the revised flaps could be saved by an early salvage revision.

Three of the six revised flaps were skin island flap designs (50%). Patients with complete flap removal had significantly longer hospitalization (p = 0.0013). The other baseline data parameter age, gender, flap indication, and comorbidity did not present any significant differences in correlation to flap survival (Table 1).

### Hyperspectral imaging

**Fig. 2:**
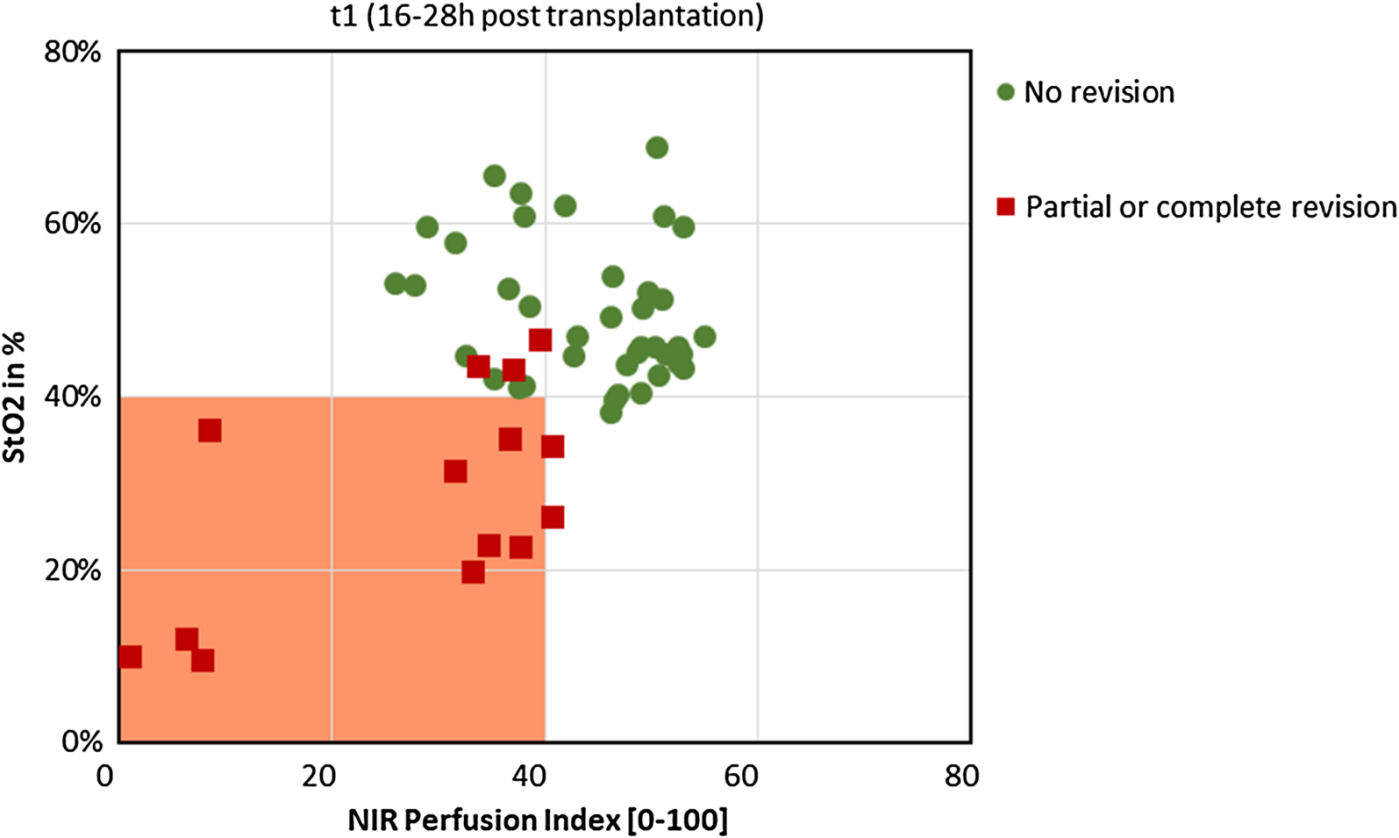
Tissue oxygenation (StO2-) and NIR Perfusion Index of all measured areas at t1 (three areas per flap). The orange square is indicating the critical zone. All areas on viable flaps were outside and at least one area of each revised flap was in the critical zone

**Fig. 3:**
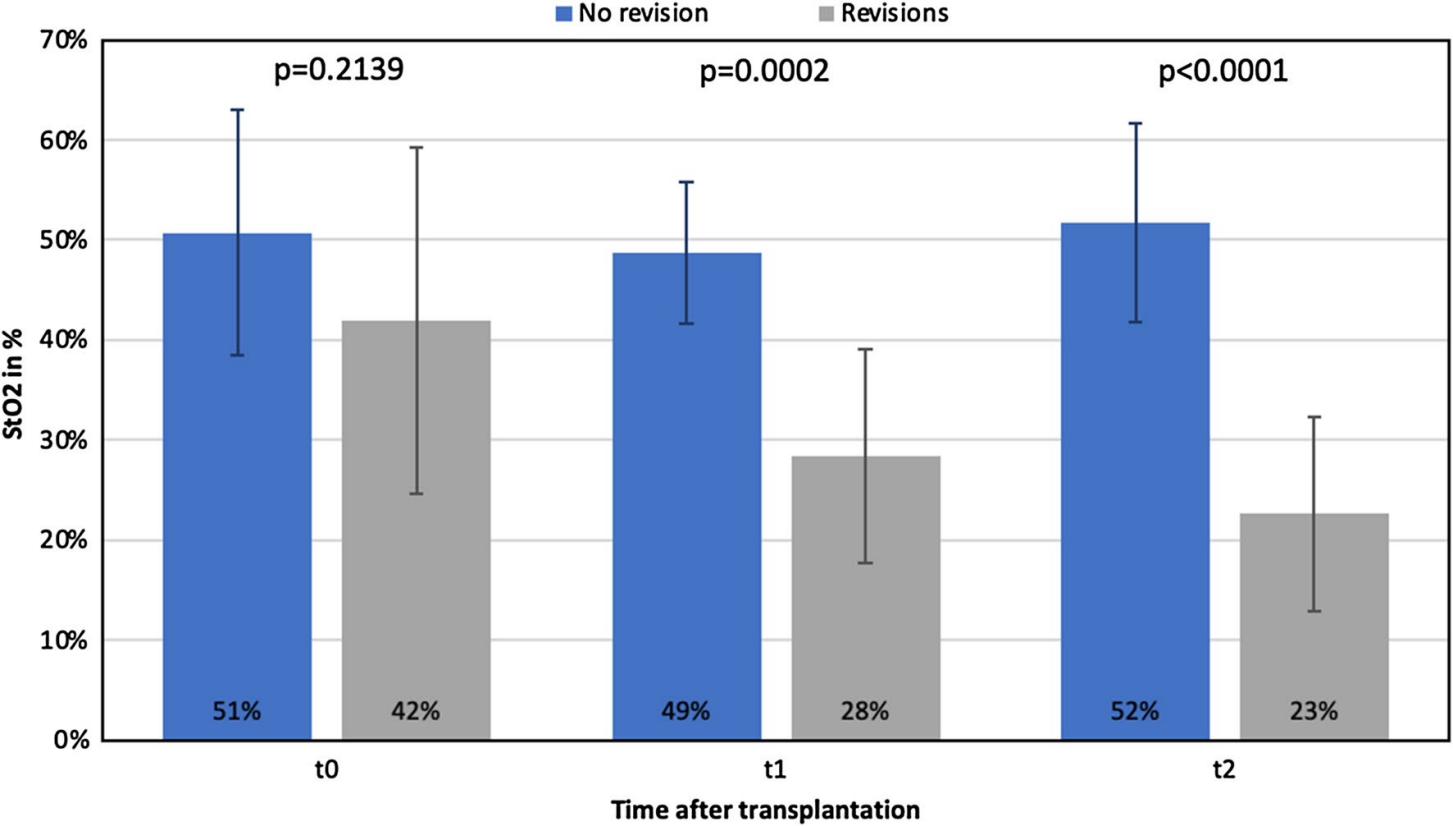
Tissue oxygenation (StO2-) Index differences over time (t0-t2) between revised (n = 6) and non-revised flaps (n = 16)

**Fig. 4:**
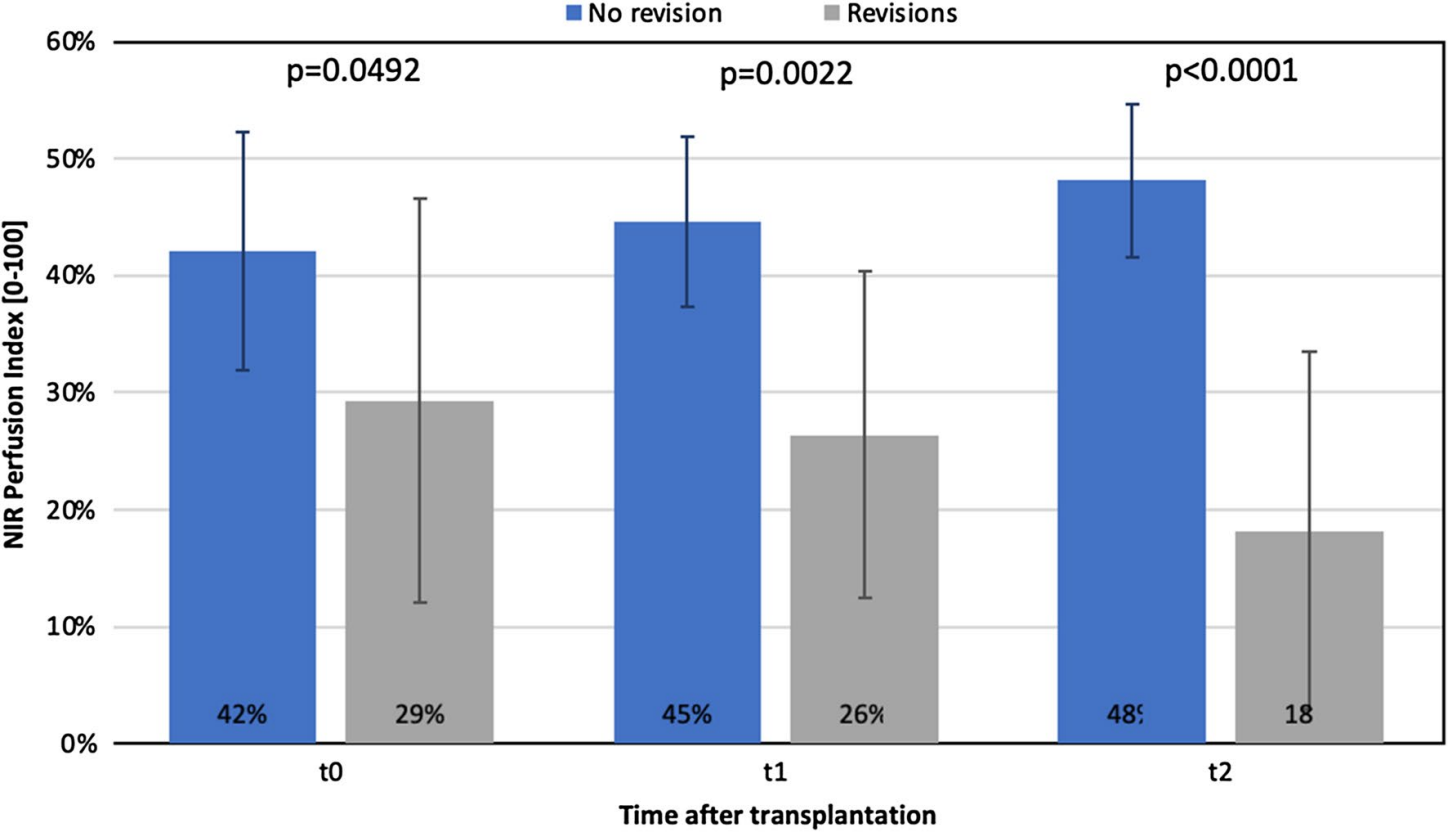
NIR Perfusion Index differences over time (t0–t2) between revised (n = 6) and non-revised flaps (n = 16)

**Fig. 5:**
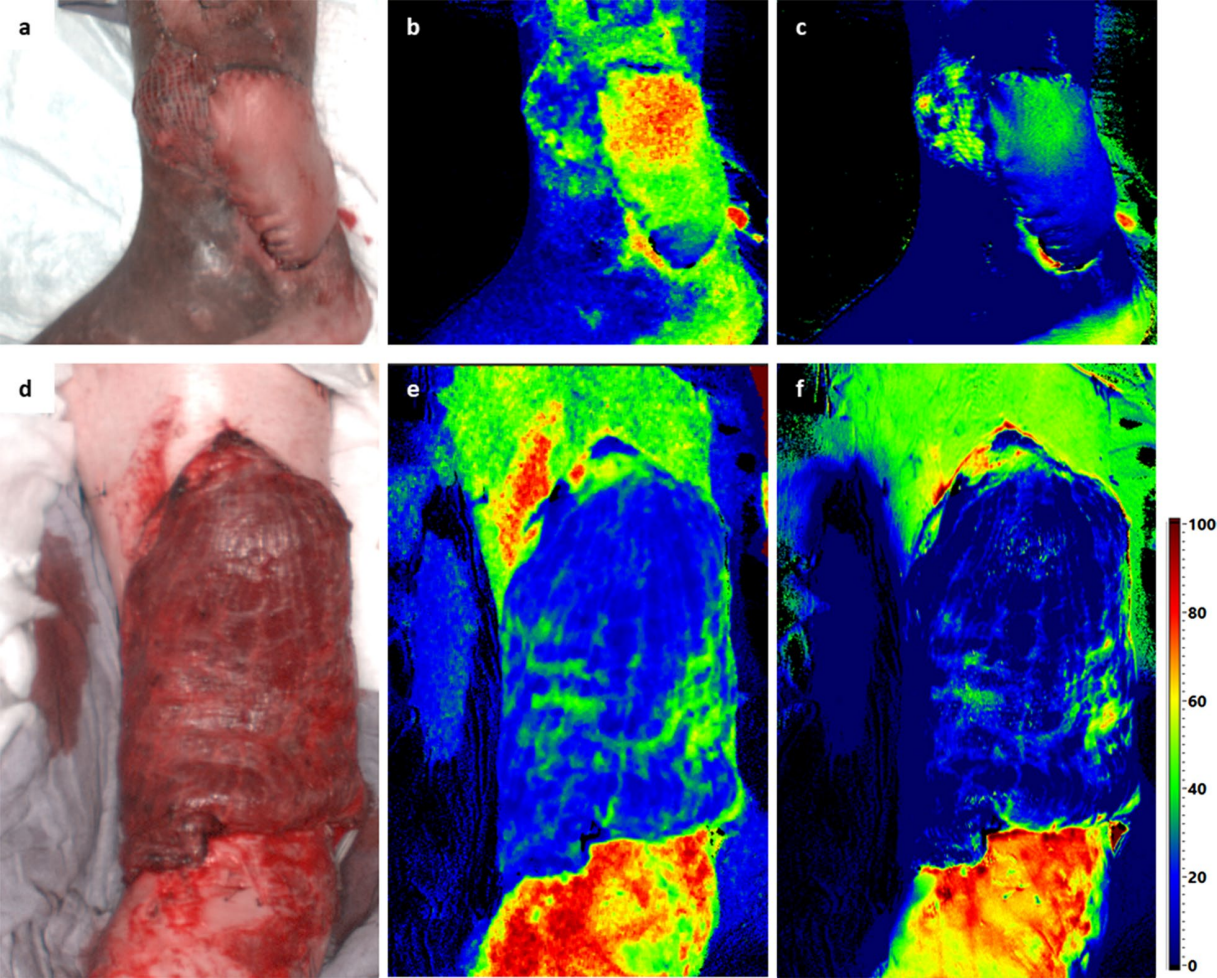
First line (**a**–**c**) showing a patients HSI results on t1 showing decreasing circulatory support with increasing distance from the A. tibialis anterior pedicle. The distal part had to be revised and covered by meshed skin graft. Second line (**d**–**f**) showing HSI results in a Latissimus Dorsi free flap tissue transfer on t0 indicating complete flap failure (both in StO2 and NIR PI)

Sixteen out of 22 flaps showed regular healing with no need for revision surgery (72.2%). In all these 16 viable flaps, both StO2 (p = 0.0002) and NIR PI (p = 0.0022) were above 40 at t1. Furthermore, all flaps that had StO2 and NIR PI values below 40 at t1 had to be partially or completely revised due to partial or complete flap loss (Fig. 2). These differences between viable (StO2 = 49; NIR PI = 45; THI = 16; TWI = 56) and revised flaps [StO2 = 28 (p < 0.001); NIR PI = 26 (p = 0.002); THI = 56 (p = 0.002); TWI = 47 (p = 0.045)] were present in all tissue parameters at t1 and even more significant (p < 0.0001 for all tissue parameters) at t2 (Figs. 3, 4). All cases with complete flap loss showed venous thrombolization. The cause of partial flap loss could not be conclusively determined. It is suspected that critical perfusion was achieved in the tissue areas furthest away from the anastomosis (Fig. 5a–c).

The HSI technique was able to provide valid data for flaps with and without skin islands as well as split skin grafted free flaps (Fig. 5d–f).

Clinical assessment and Doppler ultrasound were not able to detect flap failure at t0 or t1 in any of the revision cases. At t2, two out of 6 (25%) revised flaps showed clinical anomalies, both skin paddle free flaps. None of the three revised flaps without skin island showed clinical anomalies nor irregular Doppler ultrasound at t0–t2.

## Discussion

The monitoring of flaps in reconstructive surgery has been a much-described topic since the 1970s and is still of great interest. So far, none of the many invasive and non-invasive monitoring tools has managed to clearly distinguish itself from clinical evaluation only. Our findings indicate that hyperspectral imaging technology might close this gap, delivering reliable data in a patient and user-friendly setting.

**Fig. 6:**
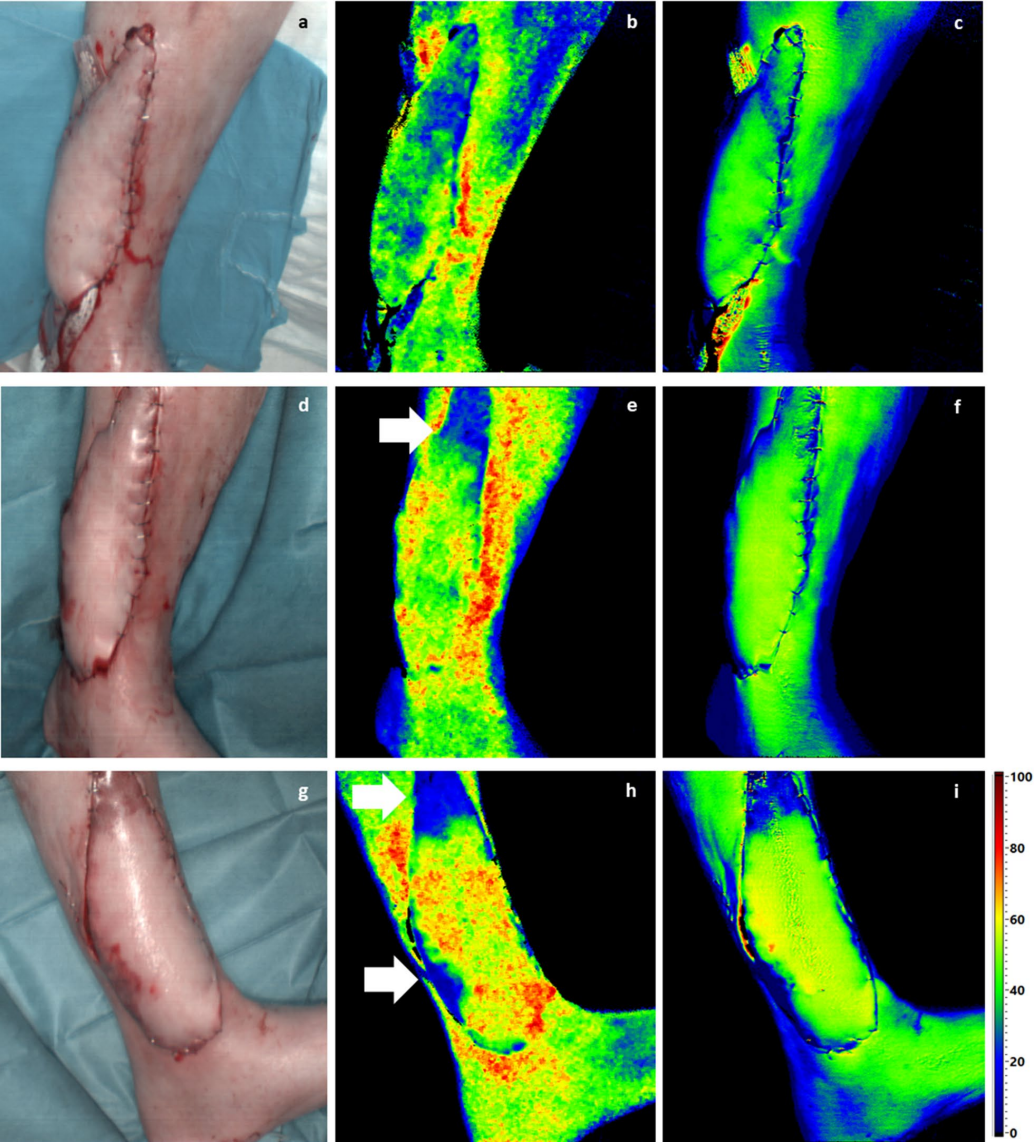
First column (**a**, **d**, **g**) = HSI color image; second column (**b**, **e**, **h**) = HSI StO2; third column (**c**, **f**, **i**) = HSI NIR Perfusion Index. First line (**a**–**c**) = t0, second row (**d**–**f**) = t1, third line (**g**–**i**) = t2. HSI photos and parameters over time. It becomes apparent that critical zones are already visible in the HSI, even though the clinical appearance is still regular (**d**–**f**). The critically areas both had to be removed and covered by meshed skin graft

In this pilot study with 22 patients, HSI was observed to demonstrate superiority to clinical and Doppler ultrasound monitoring assessments. The technique was not only accurate; it was also faster in detecting signs of vascular problems. This may help to prevent flap losses due to reasons that could be missed clinically. Chen et al. demonstrated, that the timing of occurrence of first vascular compromise signs is of the greatest importance and dictates the outcome of free flap salvage surgery [37, 39]. HSI managed to detect decreasing perfusion of the transferred tissue latest 16–28 h postoperatively. Clinical assessment and Doppler ultrasound failed to detect any of those revised cases on day 1 after surgery (Fig. 6). The Doppler ultrasound showed regular findings during the entire observation period, as the monitoring is limited to arterial feedback. Arterial thrombosis is significantly less frequent in free tissue transfer [25, 40], which also emphasizes the advantages of HSI technology in detecting venous and arterial vascular compromises. Not only does it indicate a vascular problem, but it is also able to distinguish venous from arterial compromise by pattern differentiation as Holmer et al. have already described [38].

For future decision making, the value 40 (NIR PI = 40; StO2 = 40%) seems to play a decisive role here and could be investigated as a future threshold in reconstructive surgery. So far, no cut-off values have been defined for the individual HSI parameters, but studies are outlining tissue oxygenation of 50 and higher as an indicator for regular wound healing, 30–50 as a grey zone, and lower than 30 as a predictor of bad wound healing caused by lack of perfusion [41].

Perfusion related indices have already proven to be a good indicator for vascular compromise not only in wounds [28–32] but also in reconstructive surgery. Repez et al. [42] monitored 50 flaps continuously with near-infrared spectroscopy (NIRS) and compared it to clinical observation alone. NIRS detected all cases of flow failure before clinical observation with no false positives or negatives. These results are consistent with our findings. All revised flaps could be detected earlier by HSI, especially by parameters measuring the oxygen saturation in the superficial tissue layers (StO2 up to 1 mm; NIR PI up to 4–6 mm) [38].

The limitations of this study include its small sample size and the heterogeneity of the study’s endpoint. Also, to improve selectivity in future studies with higher case numbers, it may be of interest to split individual flap entities by composition (fasciocutaneous, myocutaneous) and by the entity (e.g., ALT, latissimus dorsi, subscapular). However, we want to outline that HSI is able to analyze different tissues like skin or just muscle and is thus able to deliver reliable data for different flap designs. A cost-efficiency analysis has to be planned to not only show clinical evidence, but also practicability in the long term. The current technology allows only static images. It should be discussed whether the technique is not also capable of producing dynamic images and thus providing continuous information about perfusion conditions. Future studies may then monitor flaps continuously so that early detection of vascular compromise can allow for immediate return to the operating room. Furthermore, HSI data could help intraoperatively to analyze the perfusion of the flap after performing the anastomosis and thus help surgeons in case of early, intraoperative decision making.

Nevertheless, HSI is an approach with high potential and could lead to lower total flap failure and higher salvage rates leading to better overall survival. It combines the advantages of non-invasive and invasive monitoring methods and has so far only shown advantages compared to the other techniques.

## Conclusion

Hyperspectral Imaging combines imaging, spectroscopy and tissue oximetry in a dynamic, non-invasive and contactless fashion. It provides valuable data to monitor perfusion and oxygenation of transplanted free soft tissue flaps. The technique may detect complications at a very early stage when clinical and Doppler check-ups still have limitations due to different vascular compromise.

## Data Availability

All data is contained within the manuscript. The datasets used and analyzed during the current study available from the corresponding author on reasonable request.

## Abbreviations

HSI: Hyperspectral Imaging
et al.: Et alii. And others.
StO2: Tissue oxygenation index
NIR-PI: Near-infrared perfusion index
THI: Tissue hemoglobin index
TWI: Tissue water index
ALT: Anterolateral thigh flap
DIEP: Deep inferior epigastric perforator flap
MS-2 TRAM: Muscle sparing free transverse rectus abdominis myocutaneus flap
FOV: Field of view
SD: Standard deviation
NIRS: Near infrared spectroscopy
